# Engineering Responses to Amino Acid Substitutions in the VP0- and VP3-Coding Regions of PanAsia-1 Strains of Foot-and-Mouth Disease Virus Serotype O

**DOI:** 10.1128/JVI.02278-18

**Published:** 2019-03-21

**Authors:** Xing-Wen Bai, Hui-Fang Bao, Ping-Hua Li, Xue-Qing Ma, Pu Sun, Qi-Feng Bai, Meng Zhang, Hong Yuan, Dong-Dong Chen, Kun Li, Ying-Li Chen, Yi-Mei Cao, Yuan-Fang Fu, Jing Zhang, Dong Li, Zeng-Jun Lu, Zai-Xin Liu, Jian-Xun Luo

**Affiliations:** a State Key Laboratory of Veterinary Etiological Biology, OIE/China National Foot-and-Mouth Disease Reference Laboratory, Lanzhou Veterinary Research Institute, Chinese Academy of Agricultural Sciences, Lanzhou, Gansu, China; b Key Laboratory of Preclinical Study for New Drugs of Gansu Province, School of Basic Medical Sciences, Lanzhou University, Lanzhou, Gansu, China; University of Texas Southwestern Medical Center

**Keywords:** deleterious and compensatory effects, foot-and-mouth disease virus, genetic and phenotypic properties, PanAsia-1 strain, site-directed mutation

## Abstract

The presence of sequence divergence through adaptive mutations in the major capsid protein VP1, and also in VP0 (VP4 and VP2) and VP3, of foot-and-mouth disease virus (FMDV) is relevant to a broad range of viral characteristics. To explore the potential role of isolate-specific residues in the VP0 and VP3 coding regions of PanAsia-1 strains in genetic and phenotypic properties of FMDV, a series of recombinant full-length genomic clones were constructed using Cathay topotype infectious cDNA as the original backbone. The deleterious and compensatory effects of individual amino acid substitutions at positions 4008 and 3060 and in several different domains of VP2 illustrated that the chain-based spatial interaction patterns of VP1, VP2, and VP3 (VP1-3), as well as between the internal VP4 and the three external capsid proteins of FMDV, might contribute to the assembly of eventually viable viruses. The Y2079H site-directed mutants dramatically induced a decrease in plaque size on BHK-21 cells and viral pathogenicity in suckling mice. Remarkably, the 2079H-encoding viruses displayed a moderate increase in acid sensitivity correlated with NH_4_Cl resistance compared to the Y2079-encoding viruses. Interestingly, none of all the 16 rescued viruses were able to infect heparan sulfate-expressing CHO-K1 cells. However, viral infection in BHK-21 cells was facilitated by utilizing non-integrin-dependent, heparin-sensitive receptor(s) and replacements of four uncharged amino acids at position 3174 in VP3 of FMDV had no apparent influence on heparin affinity. These results provide particular insights into the correlation of evolutionary biology with genetic diversity in adapting populations of FMDV.

**IMPORTANCE** The sequence variation within the capsid proteins occurs frequently in the infection of susceptible tissue cultures, reflecting the high levels of genetic diversity of FMDV. A systematic study for the functional significance of isolate-specific residues in VP0 and VP3 of FMDV PanAsia-1 strains suggested that the interaction of amino acid side chains between the N terminus of VP4 and several potential domains of VP1-3 had cascading effects on the viability and developmental characteristics of progeny viruses. Y2079H in VP0 of the indicated FMDVs could affect plaque size and pathogenicity, as well as acid sensitivity correlated with NH_4_Cl resistance, whereas there was no inevitable correlation in viral plaque and acid-sensitive phenotypes. The high affinity of non-integrin-dependent FMDVs for heparin might be explained by the differences in structures of heparan sulfate proteoglycans on the surfaces of different cell lines. These results may contribute to our understanding of the distinct phenotypic properties of FMDV *in vitro* and *in vivo*.

## Abstract (precis)

The sequence variation within the capsid proteins occurs frequently in the infection of susceptible tissue cultures, reflecting the high levels of genetic diversity of FMDV. A systematic study for the functional significance of isolate-specific residues in VP0 and VP3 of FMDV PanAsia-1 strains suggested that the interaction of amino acid side chains between the N terminus of VP4 and several potential domains of VP1-3 had cascading effects on the viability and developmental characteristics of progeny viruses. Y2079H in VP0 of the indicated FMDVs could affect plaque size and pathogenicity, as well as acid sensitivity correlated with NH_4_Cl resistance, whereas there was no inevitable correlation in viral plaque and acid-sensitive phenotypes. The high affinity of non-integrin-dependent FMDVs for heparin might be explained by the differences in structures of heparan sulfate proteoglycans on the surfaces of different cell lines. These results may contribute to our understanding of the distinct phenotypic properties of FMDV *in vitro* and *in vivo*.

## INTRODUCTION

Foot-and-mouth disease virus (FMDV) is the etiological agent of an acute, highly contagious, and economically devastating vesicular disease that affects domestic and wild cloven-hoofed animals (1–3). The virus belongs to the *Aphthovirus* genus of the *Picornaviridae* family (4). Seven immunologically distinct serotypes of FMDV (O, A, C, Asia1, and SAT1 to SAT3) have been recorded (5), and multiple subtypes have continuously evolved within each serotype, reflecting significant genetic variability (6–8). The mature FMDV virion is nonenveloped, round with icosahedral symmetry, and composed of 60 copies each of four structural proteins VP1, VP2, VP3, and VP4 (VP1-4), plus a single-stranded, positive-sense RNA of approximately 8,500 nucleotides in length (9–11). The interaction of the newly transcribed genomic RNA with the myristylated capsid precursor is likely to facilitate the initiation of FMDV encapsidation for the ultimate viability of intracellular infectious viral particles (12–14). The disassembly of the FMDV capsid into pentameric subunits for viral RNA release is dependent on endosomal acidification during virus internalization (15).

As the most variable region of the FMDV genome, nucleotide sequences of the VP1 gene have been used to define genetically and geographically distinct evolutionary lineages (≥92.5%) and topotypes (≥85%), owing to its dominant role in antigenic determination (7, 16). In particular, the VP1 G–H loop of FMDV is one of the major neutralizing epitopes and an RGD (arginine-glycine-aspartic acid) motif in this domain plays a key role in integrin (αvβ1, αvβ3, αvβ6, and αvβ8)-dependent infection (17–23). The entry of field isolates into susceptible tissue cultures for efficient infection is initiated by the RGD-binding integrin activity of FMDV via clathrin-mediated endocytosis (24). However, some cell-adapted FMDVs gain the ability to utilize heparan sulfate (HS) as a receptor to facilitate viral infection (25), following the caveola-mediated endocytic pathway (26). The acquisition of the HS-binding ability was accompanied by amino acid substitutions at widely spaced locations on the outer surface of the FMDV capsid (27–35), whereas certain specific amino acid residues at these HS-related sites might lead to the formation of small plaques on cultured cells (31, 35, 36) and might be attenuated in animals (27, 33, 36–38). Previous studies have shown that acid-resistant mutants could display a reduction in plaque size (39, 40), and resistance to an acidic pH was correlated with the increased sensitivity of FMDV to the inhibition of endosomal acidification by NH_4_Cl (41–44). Therefore, it could be considered that NH_4_Cl-resistant FMDVs use integrins as their cellular receptors (41).

Numerous experiments have demonstrated that a number of individual amino acid replacements in the major capsid protein VP1 of FMDV, especially in close proximity to the RGD motif and surrounding the 5-fold symmetr*y* axis of the icosahedral virion, could affect receptor recognition (28, 31, 32, 34, 35, 37, 45–48), acid-induced disassembly (40, 42–44, 49), and viral replication and pathogenicity (37, 45, 50–52). Furthermore, in some cases, the cell-adapted phenotypes of FMDV resulted from genetic changes at or near the protomeric and pentameric interfaces and the 3-fold axis of symmetry of VP2 and VP3 (12, 27, 29, 30, 33–36, 38–41, 53–58). The conserved amino acid residues at the C terminus of the internal VP4 were also essential for maintaining viral infectivity that might be attributable to VP0 (VP4 and VP2) cleavage and RNA packaging in the stability of the synthetic FMDV provirions (13, 42, 43, 59). We have already speculated that certain specific amino acids in VP0, adjacent to or far apart from the primary substitution (L2080Q), might be responsible for the infectivity of an intergenotypic chimeric virus (rHN/TAR6-VP0) that encodes the introduced VP0 of one of the cell-adapted PanAsia-1 strains of FMDV serotype O (O/Tibet/CHA/6/99 tc [35]; see more details in Results). In this study, the compensatory effect of these unverified second-site residues in VP0 for the generation of this rescued virus was identified using site-directed mutagenesis. The functional significance in genetic and phenotypic properties of several isolate-specific residues in VP0 and VP3 of the selected PanAsia-1 viruses was then determined by plaque assays and comparison of suckling mice virulence and was examined using structural modeling. Our results help to further understand the biological implications in response to the underlying genetic diversity of viral populations during FMDV evolution *in vitro* and *in vivo*.

## RESULTS

### Deleterious and compensatory effects of amino acid mutations in VP0 and VP3 of PanAsia-1 strains for the viability of the intergenotypic chimeric FMDV derivatives.

**(i) A4008S in VP4 and several specific amino acid residues in VP2 contribute to the infectivity of an intergenotypic chimeric virus.** In our previous study, we determined that L2080Q in VP0 is crucial for HS utilization of rHN/TAR6-VP0 (an intergenotypic chimeric virus encoding the VP0 coding region of O/Tibet/CHA/6/99 tc), while the introduction of L2080Q in the VP0 coding region of rHN/FJ9-VP0 (an intergenotypic chimeric virus encoding the VP0 coding region of O/Fujian/CHA/9/99 tc) was detrimental for engineering an infectious site-directed mutant using reverse genetics techniques (35). There were three deduced amino acid differences at positions 4008, 2079, and 2136 in the VP0 coding region of the corresponding intergenotypic chimeric and site-directed mutated full-length genomic cDNA clones pOFS/TAR6-VP0 and pOFS/FJ9-VP0^L2080Q^, as well as pOFS/FJ9-VP0 and pOFS/TAR6-VP0^Q2080L^ (35).

To clarify the compensatory effect of the distinct amino acid residues in VP0 of O/Tibet/CHA/6/99 tc (comparable to that of O/Fujian/CHA/9/99 tc, see Table S1), site-directed mutations were introduced into the VP0 coding region of pOFS/TAR6-VP0 (S4008A, Y2079H, or G2136E) and pOFS/FJ9-VP0^L2080Q^ (A4008S, H2079Y, E2136G, A4008S/H2079Y, A4008S/E2136G, or H2079Y/E2136G) for the recovery of the corresponding site-directed mutants. However, none of infectious progeny viruses were rescued successfully from the transfected supernatants of nine genome-modified constructs (Table 1). These results suggested that Y2079 and two amino acid substitutions A4008S and E2136G (comparable to that of O/Tibet/CHA/6/99 wt [35]; wt, wild-type [see Table S1]) are all necessary, in the VP0 coding region of O/Tibet/CHA/6/99 tc, for maintaining the infectivity of rHN/TAR6-VP0.

**TABLE 1:** Consequences of site-directed mutations in the VP0 or VP3 coding regions of three cell-adapted PanAsia-1 strains of FMDV serotype O^*a*^

Original plasmid^*b*^	Amino acid mutation(s)^*c*^			Plaque phenotype			No.^*f*^
	VP0		VP3	Plaque size^*d*^ (BHK-21)	Virus titer (PFU/ml)^*e*^		
	VP4	VP2			BHK-21	CHO-K1	
pOFS	–	–	–	Large	(7.0 ± 2.5) × 10^7^	<5	[84, 96]
	–	–	V3174A	Large	(5.0 ± 0.5) × 10^7^	<5	31
	–	–	V3174S	Large	(5.8 ± 1.3) × 10^7^	<5	32
	–	–	V3174T	Large	(5.5 ± 0.5) × 10^7^	<5	33
pOFS/FJ5-VP0m	–	L2214F	–	/	Lethal	/	15
	A4008S	–	–	Large	(2.1 ± 0.6) × 10^7^	<5	16
pOFS/TAR6-VP0	–	–	–	Large	(7.5 ± 2.5) × 10^7^	(1.5 ± 0.5) × 10^3^	[35]
	S4008A	–	–	/	Lethal	/	1*
	–	H2065R	–	/	Lethal	/	10
	–	S2072G	–	/	Lethal	/	11
	–	C2078Y	–	/	Lethal	/	12
	–	Y2079H	–	/	Lethal	/	2*
	–	D2133N	–	/	Lethal	/	13
	–	G2136E	–	/	Lethal	/	3*
	–	K2175R	–	/	Lethal	/	14
pOFS/TAR6-VP0^Q2080L^	–	–	–	Large	(3.6 ± 1.5) × 10^7^	<5	[35]
	–	Y2079H	–	Small	(3.3 ± 2.5) × 10^7^	<5	25
	–	G2136E	–	Large	(2.6 ± 0.2) × 10^7^	<5	26
	–	Y2079H, G2136E	–	Middle	(8.0 ± 2.5) × 10^6^	<5	27
pOFS/FJ9-VP0	–	–	–	Small	(2.0 ± 0.8) × 10^7^	<5	[35]
	–	H2079Y	–	Large	(9.0 ± 1.0) × 10^6^	<5	28
	–	E2136G	–	Small	(1.1 ± 0.6) × 10^8^	<5	29
	–	H2079Y, E2136G	–	Large	(2.0 ± 0.1) × 10^7^	<5	30
pOFS/FJ9-VP0^L2080Q^	–	–	–	/	Lethal	/	[35]
	A4008S	–	–	/	Lethal	/	4
	–	H2079Y	–	/	Lethal	/	5
	–	E2136G	–	/	Lethal	/	6
	A4008S	H2079Y	–	/	Lethal	/	7*
	A4008S	E2136G	–	/	Lethal	/	8*
	–	H2079Y, E2136G	–	/	Lethal	/	9*
pOFS/FJ9-VP3	–	–	–	Large	(4.7 ± 0.2) × 10^7^	<5	17
	–	–	D3060G, A3174T	/	Lethal	/	18
	–	–	D3060G	/	Lethal	/	19
	–	–	A3174T	Large	(5.0 ± 2.5) × 10^7^	<5	20
	–	–	A3174S	Large	(5.5 ± 2.0) × 10^7^	<5	34
	–	–	A3174V	Large	(5.5 ± 3.0) × 10^7^	<5	35
pOFS/FJ9-VP423	–	–	–	Middle	(4.5 ± 1.0) × 10^7^	<5	21
	–	–	D3060G	/	Lethal	/	22
	–	–	A3174T	Middle	(5.3 ± 1.8) × 10^7^	<5	23
	–	–	D3060G, A3174T	/	Lethal	/	24

a Three Chinese cell-adapted PanAsia-1 viruses—O/Fujian/CHA/5/99 tc, O/Tibet/CHA/6/99 tc, and O/Fujian/CHA/9/99 tc—were derived from swine or bovine clinical samples after serial passages in BHK-21 cells (35, 62). rHN was rescued from BSR-T7/5 cells by the transfection of a Cathay topotype infectious cDNA (pOFS) that contains the full-length genome of O/HN/CHA/93 (84, 96, 97). –, No mutation.

b pOFS was used as the original backbone for the generation of the expectant recombinant plasmids. The construction of five intergenotypic chimeric cDNA clones—pOFS/FJ5-VP0m (=pOFS/FJ5-VP0^L2214F^), pOFS/TAR6-VP0 and pOFS/FJ9-VP0 (35), and pOFS/FJ9-VP3 and pOFS/FJ9-VP423—was performed by the exchange-cassette strategy to replace the VP0 and/or VP3 coding regions of pOFS with O/Fujian/CHA/5/99 tc (FJ5), O/Tibet/CHA/6/99 tc (TAR6), and O/Fujian/CHA/9/99 tc (FJ9), respectively (see Materials and Methods). The introduction of amino acid mutation(s) in the corresponding VP0 or VP3 coding regions was carried out to produce pOFS/TAR6-VP0^Q2080L^ and pOFS/FJ9-VP0^L2080Q^ (35), and the other 32 site-directed mutated plasmid derivatives (see Materials and Methods). All the rescued viruses should be designated by replacing the “pOFS” characters of the corresponding plasmid constructions with “rHN” in this study.

c Standard one-letter amino acid codes are used. Amino acid residues are denoted by a four-digit numbering system. The first digit represents the protein (2, VP2; 3, VP3; and 4, VP4), and the last three digits represent the amino acid position numbered independently for each protein from the N terminus to the C terminus.

*^d^*The mean plaque sizes formed on BHK-21 cells (in diameter) were scored as follows: large, >4.0 − 0.4 mm; small, ≤2.0 + 0.2; 2.0 + 0.2 < middle ≤4.0 − 0.4 mm.

*^e^*The titer of each virus was determined by plaque-forming assays at least in duplicate or more. “Lethal” and “<5” mean no plaques produced on BHK-21 and CHO-K1 cells, respectively. /, not done.

*^f^*Numbers of plasmids constructed in this study. The plasmid numbers are arranged in the order in which they appear in Results. *, The deduced amino acid sequences in the capsid protein-coding regions of no. 1 and no. 9, no. 2 and no. 8, and no. 3 and no. 7 are 100% identical to each other. The numbers in brackets are references.

To further investigate the VP0 amino acid sequences of O/Tibet/CHA/6/99 tc, the other isolate-specific residues of five PanAsia-1 viruses (see Table S1)—H2065R (O/Fujian/CHA/9/99 wt, [35]), S2072G (O/Tibet/CHA/1/99 [60]), C2078Y (O/TAW/2/99 tc [61]), D2133N (small plaque-cloned virus [SPV] of O/JPN/2000 [36]), and K2175R (O/Fujian/CHA/5/99 tc [62])—were introduced independently into the VP0 coding region of pOFS/TAR6-VP0 for the contruction of expectant recombinant plasmids. Unfortunately, it was not possible to generate infectious site-directed mutants following transfection of BSR-T7/5 cells with these linearized plasmid cDNAs and successive blind passages of the corresponding transfected supernatants in BHK-21 cells (Table 1). These results illustrated that certain conserved amino acid residues in VP0 of rHN/TAR6-VP0 are also essential for maintaining viral infectivity.

To this end, it is likely that there was a coevolution of L2080Q with A4008S and E2136G, in the fixation of some specific amino acid residues in VP0, during the adaptation of O/Tibet/CHA/6/99 tc to BHK-21 cells. Surprisingly, though, replacement of the VP0 coding region of pOFS with O/Fujian/CHA/5/99 tc (pOFS/FJ5-VP0m=pOFS/FJ5-VP0^L2214F^ [see Materials and Methods]) led to undetectable progeny virus, and this deleterious effect could be compensated for by a second-site mutation A4008S of VP4 (Table 1). There were only two deduced amino acid differences in the VP0 coding region of pOFS/FJ5-VP0m (noninfectious, A4008 and 2175R) and pOFS/TAR6-VP0^Q2080L^ (infectious, 4008S and K2175). By this token, we assumed that certain specific amino acid residues of VP2 (B–C loop, E–F loop, G–H loop, or C terminus) are very closely intercconnected with the N terminus of VP4, in the inner surface of the FMDV capsid, for the propagation of infectious progeny viruses (see Discussion).

**(ii) D3060G in VP3 is detrimental for the generation of infectious site-directed mutants.** Although the introduction of D2133N in the VP0 coding region of pOFS/TAR6-VP0 was detrimental for the recovery of the corresponding site-directed mutant (Table 1), there were only D2133N and H3056R in VP1-4 of the SPV of the O/JPN/2000 strain on primary bovine kidney cells that led to lower virulence in suckling mice (comparable to that of the LPV [large plaque-cloned virus] [36]). In this case, we generated another intergenotypic chimeric virus, rHN/FJ9-VP3, that encodes the VP3 coding region of O/Fujian/CHA/9/99 tc (Table 1). Then, D3060G and A3174T were introduced into the VP3 coding region (100% identical to LPV of O/JPN/2000; see Table S1) of the corresponding infectious cDNA pOFS/FJ9-VP3. If the expectant site-directed mutant containing the VP3 coding region of LPV of O/JPN/2000 could be rescued successfully, then the desired site-directed mutant encoding the VP3 coding region of the SPV of O/JPN/2000 strain would be constructed by introducing H3056R into this plasmid-derived region for comparative analysis of viral plaques on cultured cells and viral pathogenicity in suckling mice. Nonetheless, the transfection outcome deviated from our initial expectation (Table 1).

To determine whether one or both of D3060 and A3174 might be required for the infectivity of rHN/FJ9-VP3, single amino acid mutations (D3060G or A3174T) were introduced into the VP3 coding region of pOFS/FJ9-VP3. Meanwhile, the other intergenotypic chimeric virus, rHN/FJ9-VP423, was generated by the exchange-cassette strategy to replace both the VP0 and VP3 coding regions of pOFS with O/Fujian/CHA/9/99 tc, following transfection into BSR-T7/5 cells (Table 1). D3060G, A3174T, or D3060G/A3174T were subsequently introduced into the VP3 coding region of this infectious cDNA pOFS/FJ9-VP423. The results of transfection showed that D3060G could exert a deleterious effect for retaining the postinfectious phenotype of the respective progeny viruses and the substituted coding region of the VP0 gene of O/Fujian/CHA/9/99 tc was unable to compensate for this lethal mutaion (Table 1).

Alignment of the capsid coding sequences of the selected PanAsia-1 viruses (see Table S1), a few isolate-specific residues were fixed in VP1 to VP4 of O/JPN/2000 (LPV: 2039V, 2079H, 3060G, and 3174T [63]). 3060G was located in VP3 of O/UKG/34/2001 (PanAsia-1 [64]) and 2079H was detected in VP2 of O/UKG/34/2001 from persistently infected cattle (including rHN and O/Fujian/CHA/9/99 tc) (38). Thus, our data implied that some specific amino acid residues in VP1 of FMDV PanAsia-1 strains (see Table S1) might be responsible for the fitness of gene matching to restore the infectivity lost by the introduction of D3060G in the VP3 coding region of the indicated intergenotypic chimeric viruses.

### Biological implications of amino acid difference at position 2079 in VP0 of two intergenotypic chimeric viruses and their site-directed mutants.

**(i) An amino acid difference at position 2079 causes the change in plaque size of FMDV on BHK-21 cells.** As already previously mentioned, comparison of the size of plaques formed by rHN/FJ9-VP0, rHN/TAR6-VP0, and rHN/TAR6-VP0^Q2080L^ on BHK-21 cells has shown that the individual amino acid residues at positions 4008, 2079, and 2136, rather than 2080 in VP0, might have a potential role in the distinct plaque phenotypes of O/Tibet/CHA/6/99 tc and O/Fujian/CHA/9/99 tc (35).

**FIG 1:**
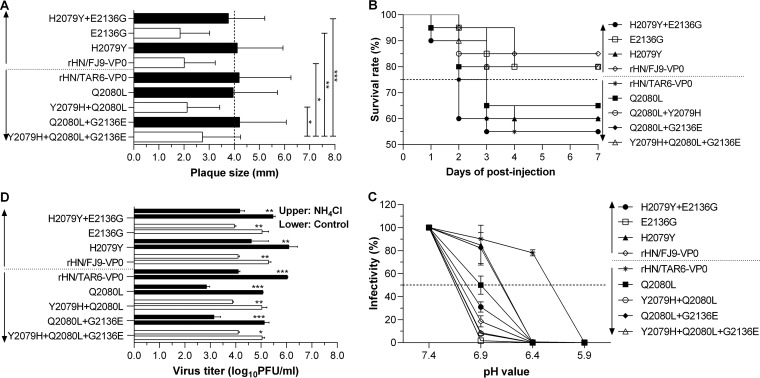
Effects of amino acid difference at position 2079 in the VP0 coding region of PanAsia-1 strains on phenotypic properties of the indicated FMDVs. rHN/TAR6-VP0, rHN/FJ9-VP0, and their seven site-directed mutants were selected to be tested by three different plaque assays on BHK-21 cells and virulence comparison in suckling mice (means ± the SD are indicated; ANOVA results are indicated [*, *P < *0.05; **, *P < *0.01; ***, *P < *0.001, if necessary]). (A) The plaques formed by the Y2079-encoding viruses (■) were distinctly larger than those formed by the 2079H-encoding viruses (□) on BHK-21 cells. At least 50 plaques were analyzed for each virus. (B) The Y2079-eoncoding viruses (●, ▲, ■, ◆, and ж) showed higher virulence in suckling mice than the 2079H-encoding viruses (□, ◇, ○, and △). A total of 20 animals were injected with each virus supernatant (about 100 PFU/200 μl per mouse). (C) The Y2079-encoding viruses (■) displayed a moderate decrease in acid-induced inactivation at acidic pH 6.9 compared to that of the 2079H-encoding viruses (□). The infectivity (%) of each diluted virus (0.5 MOI) at different pH values was calculated as the percentage of PFU by comparison with that obtained at neutral pH 7.4. (D) The infection efficiency of the Y2079-encoding viruses (●, ▲, ■, ◆, and ж) was more sensitive to NH_4_Cl treatment than that of the 2079H-encoding viruses (□, ◇, ○, and △). The total yield per virus collections in 25 mM NH_4_Cl (experimental group)- or DMSO (control group)-treated BHK-21 cells was determined by plaque-forming assay at 5 h postinfection.

To identify the functional amino acid residues in VP0 critical for the formation of different plaque morphology of the indicated FMDVs, six site-directed mutants were further rescued from BSR-T7/5 cells by tranfection of the expectant NotI-linearized plasmid cDNAs that contain amino acid mutations (2079, 2136, or 2079/2136) in the VP0 coding region of pOFS/TAR6-VP0^Q2080L^ and pOFS/FJ9-VP0 (Table 1). A plaque-forming assay was performed to measure the mean plaque sizes of rHN/TAR6-VP0, rHN/FJ9-VP0, and seven site-directed mutants (including rHN/TAR6-VP0^Q2080L^) on BHK-21 cells. The results of statistical comparison showed that amino acid replacements at position 2079 (histidine or tyrosine) could dramatically change the plaque size of these rescued viruses on BHK-21 cells (Fig. 1A). Amino acid substitutions at positions 4008 (alanine or serine) and 2136 (glutamic acid or glycine) in VP0 had some influence on the smaller size of plaques formed by the 2079H-encoding viruses (partially) but not on the Y2079-encoding viruses with large plaque morphology (Fig. 1A). Therefore, these results revealed that the individual amino acid residues at position 2079 of VP0 ought to be one of the molecular determinants for the distinct plaque phenotypes of FMDV PanAsia-1 strains *in vitro*.

**(ii) An amino acid difference at position 2079 affects the virulence of FMDV in suckling mice.** Horsington and Zhang reported that Y2079H or L2080Q might be associated with persistent infection of O/UKG/34/2001 in cattle (38). Coincidentally, amino acid differences at positions 2079 and 2080 were also found in VP2 between O/Fujian/CHA/9/99 tc and O/Tibet/CHA/6/99 tc (35; see also Table S1).

To evaluate amino acid mutations at positions 2079 and 2080 in VP0 of the indicated FMDVs in response to viral pathogenicity, two intergenotypic chimeric viruses and their seven site-directed mutants were injected into suckling mice. No major difference in virulence was observed between rHN/TAR6-VP0 and rHN/TAR6-VP0^Q2080L^; however, estimation of the survival percentages of virus-infected suckling mice showed that the Y2079-encoding viruses displayed a higher level of the virulent phenotype than the 2079H-encoding viruses (Fig. 1B). It thus appeared that the distinct amino acid residues at position 2079 in VP0 might be capable of inducing the particular capability in the infection of FMDV PanAsia-1 strains *in vivo*.

**(iii) An amino acid difference at position 2079 confers the alteration in acid-induced inactivation correlated with NH_4_Cl resistance of FMDV in BHK-21 cells.** It has been documented that (i) a tyrosine replacement of the VP2 histidine (H2145Y) could induce a decrease in acid sensitivity of C-S8c1 and Asia1/YS/CHA/2005, (ii) the acid-resistant mutants of A12 and C-S8c1 clones formed slightly smaller plaques on BHK-21 cells, and (iii) resistance to acid-induced inactivation of O/YS/CHA/2005, C-S8c1, and Asia1/YS/CHA/2005 was correlated to the increased inhibitory effect of NH_4_Cl (39–44).

To explore the effect of a histindine or tyrosine at position 2079 in VP0 on the acid sensitivity of the indicated FMDVs, nine rescued viruses were subjected to an acid-induced inactivation assay in BHK-21 cells. As a result, the Y2079-encoding viruses showed a moderate acid resistance at pH 6.9 compared to the 2079H-encoding viruses (Fig. 1C). Subsequently, the resistance of these selected FMDVs to NH_4_Cl treatment was evaluated in BHK-21 cells. As expected, infection with the Y2079-encoding viruses was more effectively inhibited by the drug (25 mM) than infection with the 2079H-encoding viruses (Fig. 1D), which further verified a correlation in acid-sensitive and NH_4_Cl-resistant phenotypes of FMDV.

Taken together, our experimental data confirmed that an amino acid difference at position 2079 in VP0 of PanAsia-1 strains might be involved in the distinct phenotypic properties of the specific FMDV variants.

### Analysis of the impact of amino acid replacements at position 3174 in VP3 of FMDV on heparin affinity.

As shown by a previous study, the occurrence of E3173K in VP3 of C-S8c1p100c10 and MARLS variant derivatives (C-S8c1, equivalent to 3174 in VP3 of FMDV serotype O) might play a role in heparin binding for FMDV infection in CHO cells (53). Here, four different uncharged amino acids (valine, alanine, serine, or threonine) were presented at residue 3174 in VP3 of the selected PanAsia-1 viruses (see Table S1).

**FIG 2:**
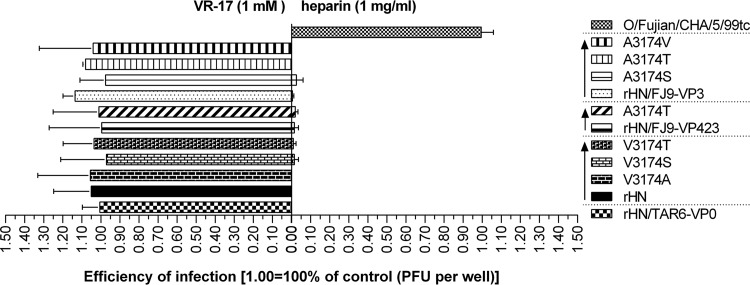
Effect of amino acid replacements at position 3174 in the VP3 coding region of the specific FMDVs for heparin affinity in BHK-21 cells. The infection of BHK-21 cells with rHN, rHN/FJ9-VP0, rHN/FJ9-VP423, and their seven site-directed mutants was examined by the RGD-containing peptide VR-17 inhibition and heparin binding assays (see Materials and Methods). O/Fujian/CHA/5/99 tc and rHN/TAR6-VP0 were used as negative and positive controls separately. The results from these two plaque assays demonstrated that the remaining plaques of each virus on BHK-21 cells seemed to have been reduced by the addition of heparin (1 mg/ml, right) rather than the preincubation of the RGD-containing peptide VR-17 (1 mM, left). The high affinity of these ten rescued viruses for heparin was not affected by four uncharged amino acid replacements at position 3174 in VP3.

To assess the influence of these specific amino acid residues at position 3174 in VP3 of FMDV serotype O on heparin binding and the infection of cells in culture, site-directed mutations were introduced into the VP3 coding region of pOFS (V3174A, V3174S, or V3174T) and pOFS/FJ9-VP3 (A3174S or A3174V) for the generation of the other five site-directed mutants (Table 1). A plaque-forming assay on CHO-K1 cells and RGD-containing peptide inhibition and heparin binding assays on BHK-21 cells were performed to determine the usage of cellular receptors and heparin affinity of rHN, rHN/FJ9-VP3, rHN/FJ9-VP423, and their seven site-directed mutants. The results from three different assays demonstrated that all of these ten rescued viruses were unable to utilize HS as a receptor for efficient infection of CHO-K1 cells (Table 1) but could almost completely dispense with their RGD integrin-binding motif and facilitate viral infection in the expression of heparin-sensitive receptor(s) on the surfaces of BHK-21 cells (Fig. 2).

According to the results presented above, it seems that the presence of these four uncharged amino acid residues at position 3174 in VP3 might have no impact on the affinity of Cathay topotype and PanAsia-1 lineage of FMDV serotype O for heparin.

## DISCUSSION

The adaptation of FMDV in susceptible host cells by sequence divergence is a continuous and dynamic process of positive selection, accompanying conservative and compensatory evolution in the progeny of viral RNA molecules (1, 6, 65–70). Reverse genetics is an extremely powerful approach for elucidating the genetic response of the adaptive mutations to biological implications of the FMDV populations (56, 57, 71). As one of the most pandemic lineages of FMDV serotype O that caused widespread outbreaks in Asia, Europe and Africa (72), it was unfortunate that no other infectious full-length cDNAs of PanAsia-1 strains have been published, except for O/YS/CHA/05 (43, 73). This may be due to the population bottlenecks of viral quasispecies (74–77).

**FIG 3:**
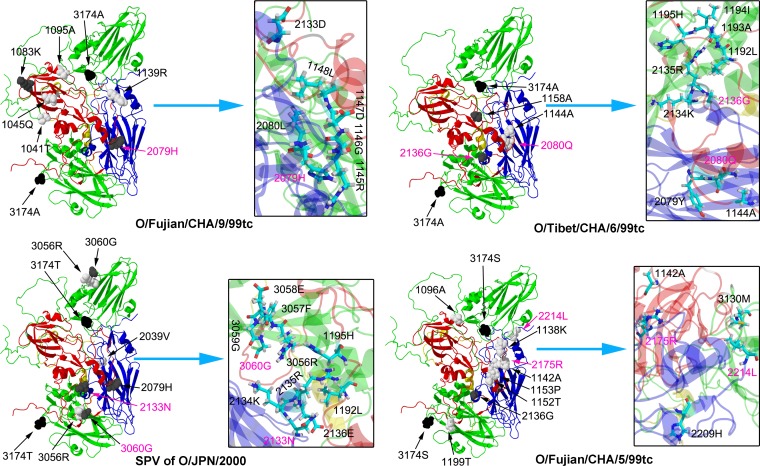
Location of isolate-specific amino acid residues in VP0 and VP3 of the icosahedral capsid of the selected PanAsia-1 strains of FMDV serotype O. The ribbon protein diagram represents 1 of the 60 protomeric subunits, plus one additional VP3 from a neighboring protomer. The structural proteins VP1 (missing 211 to 213 residues), VP2 (missing 1 to 4 residues), VP3, and VP4 (missing 1 to 14 and 41 to 64 residues) are highlighted as red, blue, green, and yellow (internal), respectively. The individual amino acid residues of VP1 to VP3 (listed in Table S1) are labeled as the space-filling atomic models. The target residues (left) of enlarged areas (right) are marked in pink font. The atomic-level interaction of isolate-specific amino acid residues was visualized, and the minimal side chain-side chain distances measured of critical amino acid residues were 4.71 Å between 2079H (N_τ_) and R1145 (N_ω_) of O/Fujian/CHA/9/99 tc, 4.68 Å between L2080Q (C_δ_) and S1144A (C_β_), 5.20 Å between E2136G (N) and L1192 (C_γ_) of O/Tibet/CHA/6/99 tc, 5.63 Å between D2133N (C_β_) and L1192 (C_δ_), 5.78 Å between 3060G (C_α_) and K2134 (C_ε_) of the SPV [small plaque-cloned virus] of O/JPN/2000 strain, 4.42 Å between 2175R (C_ε_) and 1142A (C_α_), and 3.25 Å between 2214L (C_δ_) and M3130 (C_ε_) of O/Fujian/CHA/5/99 tc. The corresponding C_α_-C_α_ distances were 7.07, 7.49, 7.33, 10.05, 9.90, 9.14, and 7.29 Å, respectively.

To date, an intergenotypic chimeric FMDV (O1K/O-UKG [50]) encoding the entire capsid proteins of O/UKG/34/2001 has been carried out for the characterization of viral pathogenicity. In our previous and present studies, four infectious intergenotypic chimeric cDNA clones (pOFS/TAR6-VP0, pOFS/FJ9-VP0, pOFS/FJ9-VP3, and pOFS/FJ9-VP423) were constructed that contain the VP0 and/or VP3 coding regions of O/Tibet/CHA/6/99 tc and O/Fujian/CHA/9/99 tc (35) (Table 1). However, site-directed mutations into the VP0 coding region of pOFS/TAR6-VP0 (H2065R, S2072G, C2078Y, Y2079H, D2133N, G2136E, or K2175R) and pOFS/FJ9-VP0 (L2080Q [35]), as well as the VP3 coding regions (D3060G) of pOFS/FJ9-VP3 and pOFS/FJ9-VP423, were detrimental for the generation of infectious progeny viruses (Table 1). Synonymous codon usage in the VP0 coding region did not give rise to any strikingly varied outcome (Table 1, partial data not shown). These individual amino acid residues were mapped in the B–C loop, the E–F loop, and the G–H loop of VP2 and in the B–B knob of VP3 of the selected PanAsia-1 viruses (see Table S1). The three-dimensional conformation of the FMDV capsid showed that (i) the VP2 B–C loop lies in the vicinity of the 3-fold symmetr*y* axis or somewhere nearby the interpentamer interfaces, (ii) the VP2 E–F loop and the VP3 B–B knob surround a central channel of the trapezoidal structures of VP1-3 in each protomer, and (iii) the VP2 G–H loop is placed on a large Y-shaped intersection of two protomeric subunits (10, 21, 58, 78, 79). The molecular dynamic stimulations revealed that seven of the eight isolate-specific residues in VP2 (excluding G2072) of the respective PanAsia-1 viruses were clustered around the positions occupied by the G–H loop or C terminus of VP1 (Fig. 3, partial data not shown). The VP1 G–H loop protrudes from the capsid outer surface, with very different stereochemical orientations depending on the specific FMDV variants (80). The VP1 C terminus of FMDV forms a long arm structure, spanning over the VP3 E–F loop of the same protomer to the VP1 G–H loop from a 5-fold related protomer (10). These two highly variable regions of VP1 strongly affect antigenic and receptor-binding properties of FMDV (30, 81). This prompted that the lethal mutations in VP2 might perturb the flexibility of the G–H loop and C terminus of VP1 for the capsid structural stability of FMDV. The measurement of minimal side chain-side chain distances reflected that G2072 (N) and D3060G (C_α_) were closest to L2187 (C_β_) in the G–H loop and K2134 (C_ε_) in the E–F loop, of VP2 of O/Tibet/CHA/1/99 and the SPV of O/JPN/2000 strain, respectively (Fig. 3, partial data not shown). It follows that the distant location of very limited sequence variations on the FMDV capsid may cause a cascading interaction by hydrogen bonding and Van der Waals forces for modulating virus genetic adaptability (27, 79). In addition, A4008S at the N terminus of VP4 was responsible for restoring the infectivity of pOFS/FJ5-VP0m and maintaining an infectious viral phenotype of pOFS/TAR6-VP0 (Table 1). The sequence comparision showed that several individual amino acid residues existed in the VP0 coding region of O/Fujian/CHA/5/99 tc (2136G, 2175R, and 2214L) and O/Tibet/CHA/6/99 tc (A4008S, L2080Q, and E2136G), respectively (35, 60; see also Table S1). It was inferred that the interaction of amino acid side chains between VP4 and VP1-3 in a functional capsid region might be involved in the conformation of flexible scaffolding in the inner capsid surface of FMDV, despite of little regular secondary structure of the highly conserved VP4 (29, 78, 82, 83).

Anyhow, we have succeeded in producing several infectious site-directed mutants for the investigation of the possible adaptive responses to amino acid differences in the VP0 coding region of O/Tibet/CHA/6/99 tc and O/Fujian/CHA/9/99 tc (35) (Table 1). In the present study, the results of plaque-forming assay and virulence determination further demonstrated that a single amino acid change at position 2079 (tyrosine or histidine) in VP0 of O/Tibet/CHA/6/99 tc and O/Fujian/CHA/9/99 tc was statistically sufficient to alter the plaque size on BHK-21 cells (Fig. 1A) and the pathogenicity in suckling mice (Fig. 1B) of the corresponding intergenotypic chimeric viruses and site-directed mutants. In addition, the 2079H-encoding viruses were more sensitive to acid-induced inactivation (Fig. 1C) correlated with NH_4_Cl resistance (Fig. 1D) than that of the Y2079-encoding viruses in BHK-21 cells, which were not exactly consistent with results from previous studies (39, 40, 44). In fact, (i) O/Tibet/CHA/6/99 tc (Y2079) displayed extreme acid sensitivity (comparable to that of the Y2079-encoding viruses), and (ii) the backbone virus (rHN, H2079) formed obviously larger plaques and induced an increase in resistance to acid-induced inactivation (comparable to that of the 2079H-encoding viruses) in the infection of BHK-21 cells (35) (Table 1, partial data not shown). In view of these data, there might be no inevitable correlation between the phenotypes of plaque morphology and acid sensitivity of FMDV and some of the individual amino acid residues in VP1-4 could definitely have cooperative effects on the distinct phenotypic properties of Cathay and PanAsia-1 viruses.

To our knowledge, the frequent capsid alterations accumulated around the G–H loop of VP1 might lead to amino acid substitutions occurring around the pore at the 5-fold axis of the icosahedrally symmetric capsid, which play an important role in the acquisition of heparin affinity of FMDV for efficient infection of CHO cells (28, 31, 32, 34, 35, 37, 45, 47, 53). Sequencing of the complete capsid coding region showed that no compenstory mutational events were noted in any of the present transfection and continuous passage experiments that yielded viable viruses. All of the 16 rescued viruses were unable to produce plaques on CHO-K1 cells (Table 1). However, infection of BHK-21 cells with these rescued viruses was inhibited efficiently by heparin rather than RGD-containing peptide, and replacements of four uncharged amino acid residues at position 3174 in VP3 had no apparent influence on high affinity for heparin (Fig. 2, partial data not shown). The fixation of specific amino acid residues (L2080M, E1083K, and D1138G of rHN [84]; see Table S1) and the minimal side chain-side chain distances between critical amino acid residues 3174 and 1135/1202 (lysine) on the FMDV capsid may be helpful for explaining some of the experimental results (35, 53) (Fig. 3). There is still one issue: what is the non-integrin-dependent, heparin-sensitive receptor(s) on the surface of BHK-21 cells? In this respect, heparin is chemically similar to HS, except for higher level of sulfation and higher content of iduronic acid (85–88). Many improtant viral entry genes are present in the genome but not expressed in the transcriptome of CHO-K1 cells, such as β4 GlcNAc (*N-acetylglucosamine*), ST6Gal (β-galactoside α2,6-sialyltransferses), and HS3ST (HS glucosamine 3-*O*-sulfotransferases), etc. (89). 3-*O*-sulfated HS serves as an entry receptor for HSV-1 (herpes simplex virus 1), and the expression of HS3ST rendered CHO-K1 cells susceptible to HSV-1 infection (90–95). This allows us to predict the difference in structure of HS proteoglycans on the surface of BHK-21 and CHO cell lines. Moreover, these valuable references would be intended to interpret the distinct phenotypic properties of the HS-binding FMDVs in different host cells (35, 37, 53). Overall, our findings may have profound implications for genetic capsid modifications of FMDV in the infection of tissue and cell cultures.

## MATERIALS AND METHODS

### Cell lines, viruses, and plasmids.

BHK-21 cells (integrins+, HS+; GDC010) were obtained from China Center for Type Culture Collection (CCTCC; Wuhan, China) and were maintained in Dulbecco modified Eagle medium (DMEM; Gibco) containing 10% fetal bovine serum (FBS; HyClone) and 2 mM l-glutamine (Gibco). CHO-K1 (integrins–, HS+; CCL-61) cells were purchased from the American Type Culture Collection (ATCC; Manassas, VA) and were grown in F-12K nutrient mixture (Gibco) supplemented with 10% FBS, 100 U/ml penicillin, and 100 μg/ml streptomycin (Sigma). BSR-T7/5 cells, a BHK-derived cell line stably expressing T7 RNA polymerase (RNAP), were kindly provided by Karl-Klaus Conzelmann (Max von Pettenkofer Institute of Virology, Munich, Germany) and were cultivated in Glasgow minimal essential medium (Gibco) added with 10% FBS, 4% tryptose phosphate broth (BD-Bacto), and 1 mg/ml G418 (Sigma). All cells in culture were incubated at 37°C in a humidified chamber with 5% CO_2_.

O/Fujian/CHA/5/99 tc, O/Tibet/CHA/6/99 tc, and O/Fujian/CHA/9/99 tc are three cell-adapted PanAsia-1 strains of FMDV serotype O (35, 62). rHN is a genetically engineered FMDV rescued from an infectious cDNA pOFS (96). rHN/TAR6-VP0, rHN/FJ9-VP0, and rHN/TAR6-VP0^Q2080L^ were generated from three infectious full-length genome-modified cDNA clones of FMDV, pOFS/TAR6-VP0, pOFS/FJ9-VP0, and pOFS/TAR6-VP0^Q2080L^, respectively (35).

pOFS containing the full-length genome of O/HN/CHA/93 (Cathay topotype [97]) was constructed by Cao et al. (84). The construction of pOFS/TAR6-VP0 and pOFS/FJ9-VP0 was performed by the exchange-cassette strategy to replace the VP0 coding region of pOFS with O/Tibet/CHA/6/99 tc (TAR6) and O/Fujian/CHA/9/99 tc (FJ9), respectively (35). Single amino acid replacements at position 2080 were then introduced into the VP0 coding region of pOFS/TAR6-VP0 and pOFS/FJ9-VP0 to produce the corresponding site-directed mutated full-length genomic cDNA clones, pOFS/TAR6-VP0^Q2080L^ and pOFS/FJ9-VP0^L2080Q^ (noninfectious [35]).

### Exchange-cassette strategy and site-directed mutagenesis.

An exchange-cassette strategy was used to produce two intergenotypic cDNA clones, pOFS/FJ9-VP3 and pOFS/FJ5-VP0m (=pOFS/FJ5-VP0^L2214F^, L2214F was introduced by the designed primers with two nucleotide mutations [gag*c*tc→gaa*t*tc, EcoRI]), by replacing the independent VP3 and VP0 coding regions of pOFS with O/Fujian/CHA/9/99 tc and O/Fujian/CHA/5/99 tc (FJ5). Replacement of both the VP0 and VP3 coding regions of pOFS with O/Fujian/CHA/9/99 tc was carried out to produce pOFS/FJ9-VP423.

The introduction of amino acid mutations in the VP0 or VP3 coding regions of pOFS, pOFS/FJ5-VP0m, pOFS/TAR6-VP0, pOFS/TAR6-VP0^Q2080L^, pOFS/FJ9-VP0, pOFS/FJ9-VP0^L2080Q^, pOFS/FJ9-VP3, and pOFS/FJ9-VP423 was performed by one-step overlap extension PCR using a QuikChange multisite-directed mutagenesis kit (Stratagene) to construct the other 32 site-directed mutated plasmids (Table 1).

A total of 35 newly constructed plasmid derivatives containing full-length genome-modified cDNAs of FMDV under the control of the bacteriophage T7 promoter were confirmed by automated sequencing (Sunnybio, Shanghai, China).

### Transfection and propagation of progeny viruses.

All of these T7 RNAP-dependent plasmid constructions were linearized by digestion with NotI (New England Biolabs) and purified using the JetQuick PCR product purification spin kit (Genomed). Subsequently, the linearized cDNAs were transfected into confluent monolayers of BSR-T7/5 cells (G418 free) in 6-well plates (Nunc) by using Lipofectamine 2000 transfection reagent (Invitrogen) according to the manufacturer’s protocol. The supernatants of transfected cells were collected once a typical cytopathic effect (CPE; ≥90%) appeared or in the absence of a visible CPE at 72 h posttransfection and then serially passaged in BHK-21 cells at least six times, each time following three freeze-thaw cycles. Finally, the genetic and phenotypic properties of the resulting virus stocks were further characterized in duplicate or more with the following experiments.

### Plaque assays. (i) Plaque-forming assays on BHK-21 and CHO-K1 cells.

BHK-21 and CHO-K1 cells were seeded in 6-well plates. Serial 10-fold dilutions of the progeny viruses from the supernatants of infected cell cultures were prepared in DMEM. Portions (200 μl) of the sample preparations of each virus suspension were inoculated onto confluent cell monolayers. After 1 h of incubation at 37°C in 5% CO_2_, the virus inoculum was removed, a 2-ml overlay medium containing 0.6% gum tragacanth (MP Biomedicals) and 1% FBS was added, and the cells were cultured for up to 48 h (BHK-21 [62]) or 72 h (CHO-K1 [34]) under the same conditions. Subsequently, cultured cells were washed three times with PBS (pH = 7.4) and fixed with 1:1 cold acetone-methanol (V/V) for 20 min at –20°C. The formation of viral plaques was viewed by staining with 0.2% crystal violet (Sigma) for 30 min at room temperature. The titer and plaque morphology of each virus were estimated by counting the plaque numbers and were analyzed by measuring the mean sizes of the plaques (large, >4.0 − 0.4 mm; small, ≤2.0 + 0.2; 2.0 + 0.2 < middle ≤4.0 − 0.4 mm [in diameter]), respectively.

### (ii) RGD-containing peptide inhibition assay on BHK-21 cells.

Cell monolayers were incubated in the presence of 10*^x^* (*x* = –4 ∼ 0) mM of the RGD-containing peptide (VR-17, 141-VPNLRGDLQVLAQKVAR-157 [46]; Invitrogen) dissolved in phosphate-buffered saline (PBS) containing 1 mM CaCl_2_ and 0.5 mM MgCl_2_ for 45 min at 37°C. The appropriate virus concentrations (10 to 50 PFU/well [35]) were then added to BHK-21 cells. The inhibition of FMDV infection by VR-17 was calculated compared to the numbers of plaques formed by each virus on the infected cells in the absence of RGD-containing peptide competition.

### (iii) Heparin binding assay on BHK-21 cells.

Each of the diluted viruses (10 to 50 PFU/100 μl [35]) was premixed with soluble heparin sodium (2*^x^* mg/100 μl, *x* = –4 ∼ 0; Sigma). After 10 min of neutralization at room temperature, the percent plaque reduction of the mixtures was determined by a plaque-forming assay on BHK-21 cell monolayers (35, 62).

### (iv) Acid-induced inactivation assay in BHK-21 cells.

Portions (10 μl) of the indicated virus stocks (0.5 multiplicity of infection [MOI]) were mixed with 300 μl of PBS solutions (50 mM NaH_2_PO_4_ and 140 mM NaCl) of different pH values (5.9, 6.4, 6.9, or 7.4) and then incubated for 30 min at room temperature. The mixtures were neutralized by the addition of 100 μl of a 1 M Tris solution (pH 7.6; Sigma), and the remaining viral particles were determined by a standard plaque assay on BHK-21 cells (41, 42, 59).

### (v) Endosomal-acidification blockage assay in BHK-21 cells.

A previously described procedure was performed, with the following minor modification (98, 99). Monolayers of BHK-21 cells in 6-well plates were pretreated for 1 h with 25 or 50 mM NH_4_Cl (Merck) in culture medium supplemented with 25 mM HEPES (pH 7.4; Gibco), and the drug was maintained throughout the rest of the assay to avoid cellular recovery. The titers of the harvested viruses at 5 h postinfection (adsorption for 1 h at 4°C and penetration for 4 h at 37°C) were developed as PFU/ml, and the decreased infectivity was calculated by comparison to that obtained from control cells treated with the same amount of dimethyl sulfoxide (DMSO; Sigma).

### Virulence analysis in suckling mice.

The concentrations of the specific viruses (100 PFU, a modification of a dosage previously described [36, 43]) were suspended in 200 μl of PBS added with 100 U/ml penicillin and 100 μg/ml streptomycin and then injected subcutaneously into 3-day-old suckling mice (Lanzhou Bio-Pharmaceutical Factory, China Animal Husbandry Industry Co., Ltd.). The general conditions of the mice were carefully monitored daily, and observations were recorded for mortality over 7 days. All experiments were conducted in compliance with the guidelines of Gansu Ethical Review Committee (license SYXK-GAN-2014-003) for the care and use of laboratory animals.

### Statistical analysis.

Analysis of variance (ANOVA) was performed using GraphPad Prism 5 (GraphPad, San Diego, CA). The data are presented as means ± the standard deviations (SD). Statistically significant differences are denoted by asterisks (*, *P < *0.05; **, *P < *0.01; ***, *P < *0.001).

## Supplementary Material

Supplemental file 1
