# Supplementary material for: Engineering Responses to Amino Acid Substitutions in the VP0- and VP3-Coding Regions of PanAsia-1 Strains of Foot-and-Mouth Disease Virus Serotype O
Source: J Virol. 2019 Mar 21;93(7):e02278-18. doi: 10.1128/JVI.02278-18 (PMC6430551; doi:10.1128/JVI.02278-18)
Supplement: Supplemental file 1 [file JVI.02278-18-s0001.pdf]

TABLES1 Alignment of the deduced amino acid sequences of the capsid protein-coding regions of PanAsia-1 lineage (upper) and Cathay topotype (lower) of FMDV serotype O[a]

FMDV[b]	VP4→VP2→
O/Tibet/CHA/6/99wt[35]	GAGQSSPATGSQNQSGNTGSIINNYYMQQYQNSMDTQLGDNAISGGSNEGSTDTTSTHTTNTQNNDWFSKLASSAFSGLFGALLA DKKTEETTLLED
O/Tibet/CHA/6/99tc[35]	.......S... ....
O/Fujian/CHA/9/99wt[35]	....
O/Fujian/CHA/9/99tc[35]	....
O/Tibet/CHA/1/99(AF506822)	....
O/TAW/2/99wt(AJ539137)	....
O/TAW/2/99tc(AJ539136)	....
O/Fujian/CHA/5/99tc(HQ009509)	....
O/YS/CHA/2005(HM008917)	....
O/JPN/2000(AB079061)	....
O/UKG/34/2001(KJ831678)	....
O/UKG/35/2001(AJ539141)	....
O/HN/CHA/93tc[97]	.......T...N............N..L......... ....
rHN[84,96]	.......T...N............N..L......... ....
	Th epitope

FMDV[b]	A1 A2 αZ B C αA D
O/Tibet/CHA/6/99wt[35]	RILTTRNGHTTSTTQSSVGVTYGYATAEDFVSGPNTSGLETRVVQAERFFKTHLFDWVTSDPFGRCYLLELPTDHKGVYGSLTDSYAYMRNGWDVEVT
O/Tibet/CHA/6/99tc[35]	....Q..............................
O/Fujian/CHA/9/99wt[35]	....R.............H...............................
O/Fujian/CHA/9/99tc[35]	....H...............................
O/Tibet/CHA/1/99(AF506822)	....G......................................
O/TAW/2/99wt(AJ539137)	....
O/TAW/2/99tc(AJ539136)	....Y................................
O/Fujian/CHA/5/99tc(HQ009509)	....
O/YS/CHA/2005(HM008917)	....
O/JPN/2000(AB079061)	..........................V.......................................H...............................
O/UKG/34/2001(KJ831678)	....
O/UKG/35/2001(AJ539141)	....
O/HN/CHA/93tc[97]	....H...............................
rHN[84,96]	....HM..............................
	Antigenic site 2-1

Continued-1

```
FMDV[b]                                   E                 αB     F         G1           G2          H                   I
O/Tibet/CHA/6/99wt[35]         AVGNQFNGGCLLVAMVPELCSIDKRELYQLTLFPHQFINPRTNMTAHITVPFVGVNRYDQYKVHKPWTLVVMVVAPLTVNTEGAPQIKVYANIAPTNV
O/Tibet/CHA/6/99tc[35]         .........................G........................................................................
O/Fujian/CHA/9/99wt[35]        ..................................................................................................
O/Fujian/CHA/9/99tc[35]        ..................................................................................................
O/Tibet/CHA/1/99(AF506822)     ..................................................................................................
O/TAW/2/99wt(AJ539137)         ..................................................................................................
O/TAW/2/99tc(AJ539136)         ..................................................................................................
O/Fujian/CHA/5/99tc(HQ009509)  .........................G......................................R.................................
O/YS/CHA/2005(HM008917)        .........................G......................................R.................................
O/JPN/2000(AB079061)           ..................................................................................................
O/UKG/34/2001(KJ831678)        ..................................................................................................
O/UKG/35/2001(AJ539141)        ..................................................................................................
O/HN/CHA/93tc[97]              ......................N............................Y............................N.................
rHN[84,96]                     ......................N............................Y............................N.................
                                                  Antigenic site 2-2

FMDV[b]                                   VP3→αZ       B          B           C
O/Tibet/CHA/6/99wt[35]         HVAGEFPSKE GIFPVACSDGYGGLVTTDPKTADPAYGKVFNPPRNMLPGRFTNFLDVAEACPTFLHFEGDVPYVTTKTDSDRVLAQFDLSLAAKHMS
O/Tibet/CHA/6/99tc[35]         .................................................................................................
O/Fujian/CHA/9/99wt[35]        .................................................................................................
O/Fujian/CHA/9/99tc[35]        .................................................................................................
O/Tibet/CHA/1/99(AF506822)     .................................................................................................
O/TAW/2/99wt(AJ539137)         .................................................................................................
O/TAW/2/99tc(AJ539136)         .................................................................................................
O/Fujian/CHA/5/99tc(HQ009509)  .....L...........................................................................................
O/YS/CHA/2005(HM008917)        .....L...........................................................................................
O/JPN/2000(AB079061)           .....................................................................G...........................
O/UKG/34/2001(KJ831678)        .................................................................R...G...........................
O/UKG/35/2001(AJ539141)        .....................................................................G...........................
O/HN/CHA/93tc[97]              Y......... ........................V..........L.......L...........................................
rHN[84,96]                     Y......... ........................V..........L.......L...........................................
                               Antigenic site 4
```

Continued-2

FMDV[b]	αA D E αB F G_1 G_2
O/Tibet/CHA/6/99wt[35]	NTFLAGLAQYYTQYSGTINLHFMFTGPTDAKARYMIAYAPPGMEPPKTPEAAAHCIHAEWDTGLNSKFTFSIPYLSAADYAYTASDAAETTNVQGWVC
O/Tibet/CHA/6/99tc[35]	....
O/Fujian/CHA/9/99wt[35]	....
O/Fujian/CHA/9/99tc[35]	....
O/Tibet/CHA/1/99(AF506822)	....
O/TAW/2/99wt(AJ539137)	....V...........
O/TAW/2/99tc(AJ539136)	....V...........
O/Fujian/CHA/5/99tc(HQ009509)	....S...........
O/YS/CHA/2005(HM008917)	....S...........
O/JPN/2000(AB079061)	....T...........
O/UKG/34/2001(KJ831678)	....
O/UKG/35/2001(AJ539141)	....
O/HN/CHA/93tc[97]	....V...........
rHN[84,96]	....V...........

FMDV[b]	H I	VP1→αZ B C
O/Tibet/CHA/6/99wt[35]	LFQITHGKADGDALVVLASAGKDFELRLPVDARTQ	TTSTGESADPVTATVENYGGETQVQRRQHTDVSFILDRFVKVTPKDQINVLDLMQTPAHTLV
O/Tibet/CHA/6/99tc[35]	....	....
O/Fujian/CHA/9/99wt[35]	....	....
O/Fujian/CHA/9/99tc[35]	....	....T...Q.................
O/Tibet/CHA/1/99(AF506822)	....	....
O/TAW/2/99wt(AJ539137)	....	....V........
O/TAW/2/99tc(AJ539136)	....	....V........
O/Fujian/CHA/5/99tc(HQ009509)	....	....
O/YS/CHA/2005(HM008917)	....	....
O/JPN/2000(AB079061)	....	....
O/UKG/34/2001(KJ831678)	....	...A..
O/UKG/35/2001(AJ539141)	....	...A..
O/HN/CHA/93tc[97]	..............I.........D..........	...A...........................I.......................I......
rHN[84,96]	..............I.........D..........	...A...........................I.......................I......

Antigenic site3

Continued-3

FMDV[b]	αA D E F G_1 G_2 Antigenic site 5
O/Tibet/CHA/6/99wt[35]	GALLRTATYYFADLEVAVKHEGNLTWVPNGAPETALDNTTNPTAYHKAPLTRLALPYTAPHRVLATVYNGNCKYGESPVTNVRGDLQVLAQKAARTLP
O/Tibet/CHA/6/99tc[35]	....A.............A..
O/Fujian/CHA/9/99wt[35]	....
O/Fujian/CHA/9/99tc[35]	....................K...R.....................
O/Tibet/CHA/1/99(AF506822)	....D.......................
O/TAW/2/99wt(AJ539137)	....
O/TAW/2/99tc(AJ539136)	....
O/Fujian/CHA/5/99tc(HQ009509)	.................................A...K...A.........TP.......
O/YS/CHA/2005(HM008917)	.................................A...K...A.A.......TP.......
O/JPN/2000(AB079061)	....
O/UKG/34/2001(KJ831678)	....
O/UKG/35/2001(AJ539141)	....
O/HN/CHA/93tc[97]	...........S...L....K.D...S...SDAR.S.............E.A..
rHN[84,96]	...........S...L....K.D...S...SGAR.S.............E.A..
	Antigenic site 1-1

FMDV[b]	H I Antigenic site 1-2
O/Tibet/CHA/6/99wt[35]	TSFNYGAIKATRVTELLYRMKRAETYCPRPLLAIHPSEARHKQKIVAPVKQLL
O/Tibet/CHA/6/99tc[35]	....
O/Fujian/CHA/9/99wt[35]	....
O/Fujian/CHA/9/99tc[35]	....
O/Tibet/CHA/1/99(AF506822)	.......V...
O/TAW/2/99wt(AJ539137)	.....................................K...............
O/TAW/2/99tc(AJ539136)	....
O/Fujian/CHA/5/99tc(HQ009509)	......................................T............S.
O/YS/CHA/2005(HM008917)	......................................T..............
O/JPN/2000(AB079061)	....
O/UKG/34/2001(KJ831678)	....
O/UKG/35/2001(AJ539141)	....
O/HN/CHA/93tc[97]	..................................Q..D..........A....
rHN[84,96]	..................................Q..D..........A....

[a]The secondary structure and five antigenic sites of FMDV assignments are as described in references [10, 20, 21,65, 82].α-helices and β-strands are represented as light and medium grey boxes, respectively.

[b]wt=wild-type, the animal-derived isolates; tc=tissue culture, the cell-adapted strains. rHN is a genetically engineered virus rescued frompOFS (a Cathay topotype infectious cDNA, containing the full-length genome of O/HN/CHA/93; [84,96, 97]).

[illegible] O/Tibet/CHA/6/99tc had a mixture of leucine and serine residues at position 1212 of VP1 and a mixture of codons foralanine and glutamic acidwas shown at position 1095 in VP1 of O/Fujian/CHA/9/99tc (35).The SPV (small plaque-cloned virus) of O/JPN/2000 straindisplayedD2133N in VP2 and H3056R in VP3 (comparable to that of the LPV [large plaque-cloned virus], [36]). Y2079H or L2080Q in VP2 of O/UKG/34/2001 was observed in the oesophageal-pharyngeal fluid (probang) samples collected from persistently infected cattle (38, 64).
